# Choroidal Morphology on Ultra-Widefield Indocyanine Green Angiography and Response to Aflibercept in Pachychoroid Neovasculopathy

**DOI:** 10.3390/ph16010073

**Published:** 2023-01-03

**Authors:** Su Yeon Han, Seung Hoon Lee, Phil-kyu Lee, Ho Ra, Jiwon Baek

**Affiliations:** 1 Department of Ophthalmology, Bucheon St. Mary’s Hospital, College of Medicine, The Catholic University of Korea, Bucheon 14647, Gyeonggi-do, Republic of Korea; 2 Department of Ophthalmology, College of Medicine, The Catholic University of Korea, Seoul 07345, Republic of Korea

**Keywords:** choroid, pachychoroid, ultra–widefield angiography, intervortex anastomosis, indocyanine green angiography

## Abstract

Purpose: This study assessed the relationship between the choroidal morphology and short-term response to aflibercept treatment in pachychoroid neovasculopathy (PNV). Methods: This was a retrospective case-control study. Ultra-widefield indocyanine green angiography (UWICGA) and optical coherence tomography (OCT) images of 90 PNV eyes of 90 patients treated with aflibercept were enrolled. Responsiveness to aflibercept was defined as a complete resolution of sub- or intra-retinal fluid after three loading doses (50 dry and 40 non-dry eyes). Subfoveal choroidal thickness (SFCT) was measured on OCT images, and choroidal vessel density (CVD), CVD asymmetry, intervortex anastomosis, and choroidal vascular hyperpermeability (CVH) were assessed on UWICGA images. Results: CVD on UWICGA differed between groups in terms of the total area (0.323 ± 0.034 in dry vs. 0.286 ± 0.038 in non-dry, *p* < 0.001) and area of each quadrant (superotemporal: 0.317 ± 0.040 vs. 0.283 ± 0.040, superonasal: 0.334 ± 0.040 vs. 0.293 ± 0.045, inferonasal: 0.306 ± 0.051 vs. 0.278 ± 0.052, inferotemporal: 0.334 ± 0.047 vs. 0.290 ± 0.046; all *p* ≤ 0.010). The CVH grade differed between groups (mean 1.480 ± 0.735 vs. 1.875 ± 0.822, *p* = 0.013). ST and IT intervortex anastomoses were common in the dry group, while SN, ST, and IT were most common in the non-dry group (*p* = 0.001). Conclusions: A poor short-term response to aflibercept treatment in PNV eyes was associated with a lower Haller vessel density, higher CVH grade, and intervortex anastomosis involving more quadrants on UWICGA.

## 1. Introduction

Type 1 neovascularization that is associated with choroidal thickening and/or dilated Haller vessels in the absence of characteristic age-related macular degeneration features such as typical drusen has been termed pachychoroid neovasculopathy (PNV) [1]. Following the concept of the pachychoroid initially suggested by Warrow and Freund, PNV shares common features and a pathophysiology related to the peculiar phenotype of choroid seen in so-called ‘pachychoroid spectrum disorders’ [2,3].

The response of PNV to anti-vascular endothelial growth factor (VEGF) treatments has been reported in numerous studies [4,5,6]. Most studies revealed that the efficacy of anti-VEGF in PNV was similar to that in neovascular age-related macular degeneration (nAMD), with a few differences such as the injection number and the disease-free period [6,7]. Considering the morphological difference between PNV and nAMD, pathophysiological differences should be expected, and the differences in the response to certain treatments is understandable [7,8,9,10].

Aflibercept is a good treatment option for PNV and polypoidal choroidal vasculopathy (PCV) with pachychoroid features (or PNV with aneurysms) [4,5,6,11]. However, a limited number of studies have assessed prognostic factors after aflibercept treatment among PNV eyes. We hypothesized that the response to aflibercept might be associated with choroidal vascular characteristics in PNV eyes. As in vivo choroidal vascular morphology can be assessed with optical coherence tomography (OCT) and ultra-widefield indocyanine green angiography (UWICGA), we analyzed the relationship between the response to aflibercept and choroidal vessel morphology on OCT and UWICGA.

## 2. Methods

This retrospective case-control study was based on a chart review and was approved by the Institutional Review Board of Bucheon St. Mary’s Hospital (no. HC21RASI0007), which waived the need for written informed consent due to the study’s retrospective design. The study was conducted in accordance with the tenets of the Declaration of Helsinki.

### 2.1. Subjects

Consecutive patients diagnosed with PNV who underwent three loading doses of aflibercept between April 2016 and April 2022 were enrolled in this study. The criteria for diagnosis of PNV were as follows: (1) Presence of choroidal neovascularization (CNV); (2) subfoveal choroidal thickness ≥ 300 μm in the diseased eye; (3) absence of typical soft or hard drusen or presence of pachydrusens only (i.e., drusenoid lesions > 125 μm that are solitary and distinct from the typical soft drusen of nAMD) [12]; (4) pachychoroid characteristics (i.e., choroidal vascular hyperpermeability (CVH), retinal pigment epithelium (RPE) abnormality independent of CNV lesions, the presence of thickened choroid with dilated choroidal vessels below the CNV, and/or a history of central serous chorioretinopathy).

The exclusion criteria were: (1) follow-up < 4 months; (2) concurrent macular disease or scar other than PNV; (3) any history of treatment for PNV (e.g., photodynamic therapy, laser photocoagulation, or intraocular injections); and (4) high myopia (≥6.00 diopters or axial length > 26 mm).

The best-corrected visual acuity (BCVA) and line and volume scan high-definition OCT (Cirrus 4000 or Cirrus 6000; Carl Zeiss Meditec, Jena, Germany) using enhanced-depth imaging were collected at baseline and after three loading injections of aflibercept. Mydriatic UWICGA images (Optos California P200DTx icg; Optos, Dunfermline, UK) were collected at baseline. From medical records, age, and sex, the laterality of the diseased eye and the presence of diabetes and hypertension were assessed.

### 2.2. Treatment and Assessment Schedule

All patients received three monthly intravitreal injections of 2 mg aflibercept (Eylea; Bayer HealthCare, Berlin, Germany). Each patient was scheduled for a follow-up examination after the initial three injections, and sub- or intra-retinal fluid was assessed at that time. Patients who showed complete fluid absorption were grouped into the dry group, and patients with persistent fluid were grouped into the non-dry group.

### 2.3. Imaging Analysis

**Figure 1:**
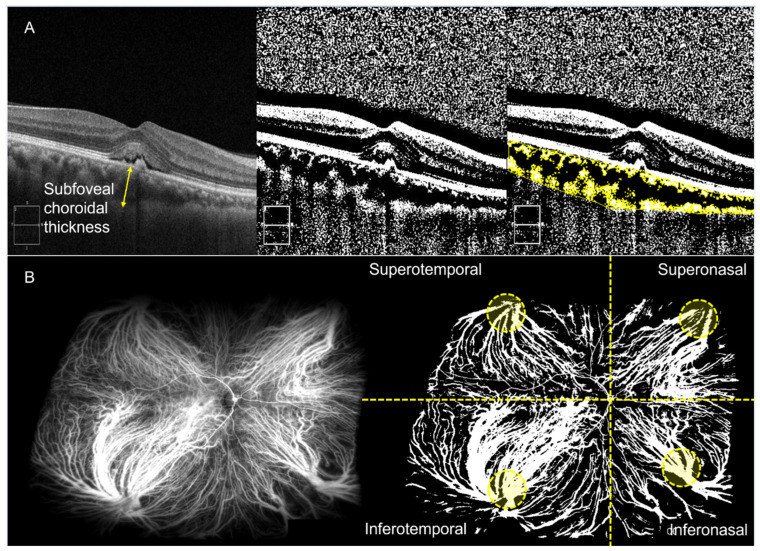
Imaging analysis with ultra-widefield indocyanine angiography (UWICGA). (**A**) Analysis of optical coherence tomography (OCT) B-scan image intersecting the center of the fovea. a. Subfoveal choroidal thickness was measured as the distance between Bruch’s membrane and the choroid–scleral border at the fovea (yellow arrow). The OCT image was binarized using “Niblack” thresholding, and the choroidal vessel density (CVD) was measured as the ratio of vascular area (black pixels) to stromal area (white pixels). (**B**) Analysis of UWICGA images. UWICGA image was binarized using “MidGrey” thresholding. CVD was calculated by dividing the number of pixels in the vascular area (white pixels) by that of the total measurable retinal area (blue dash area). Vessel densities were measured for total area, each quadrant, and each ampulla area of the quadrant.

The central macular thickness (CMT) was automatically measured in a 1-mm circle centered on the fovea using Zeiss OCT review software (version 11.5, Carl Zeiss Meditec). From the OCT horizontal B-scan intersecting the center of the fovea, the subfoveal choroidal thickness (SFCT) was measured. SFCT was defined as the distance between Bruch’s membrane and the choroid–scleral border at the fovea [13]. The same scan was binarized using “Niblack” thresholding, and the choroidal vessel density (CVD) was measured as the ratio of the vascular area (black pixels) to stromal area (white pixels) (Figure 1A).

From UWICGA images, CVD was measured based on the published method, with a slight modification [14]. The best UWICGA image, obtained between 20 s and 2 min after dye injection, was binarized using “MidGrey” thresholding, as this method better visualized large vessels. The CVD was calculated by dividing the number of pixels in the vascular area (white pixels) by that of the total measurable retinal area. Image processing and calculation were performed using MATLAB 2022a (MathWorks, Inc., Natick, MA, USA) and the FIJI software program (A distribution of ImageJ that includes plugins; U.S. National Institutes of Health, Bethesda, MD, USA; available at http://fiji.sc). Vessel densities were measured for total area, each quadrant (superotemporal (ST), superonasal (SN), inferonasal (IN), and inferotemporal (IT)), and each vortex ampulla area of the quadrant on UWICGA images (Figure 1B).

Then, CVD asymmetry was measured as follows: (1) horizontal asymmetry = CVD of (ST + SN)/(IT+IN); (2) vertical asymmetry = CVD of (ST + IT)/(SN + IN). Presence of intervortex anastomosis in the temporal and nasal retina and the dominant vessel in the macular area were assessed on UWICGA images. Anastomosis between two vortex systems was considered to be present if there were two or more anastomotic vessels connecting adjacent quadrants of vortex veins [15]. Choroidal vascular hyperpermeability (CVH) was considered as present when hyperfluorescence with blurring of vascular margins was detected during the mid-phase (>6 min) of UWICGA. CVH was grade as none (0), mild (1), moderate (2), and severe (3).

### 2.4. Statistical Analysis

Statistical analysis was performed using SPSS version 22.0.1 for Windows (IBM Corp., Armonk, NY, USA). An independent *t*-test was used to compare continuous variables between groups. When normal distribution could not be confirmed, a Mann–Whitney *U* test was used. A chi-square test was used for categorical variables between groups, and Pearson’s correlation coefficient was incorporated for correlation analysis. A paired *t*-test was used to compare the CVDs of each quadrant. Continuous variables are described as mean ± standard deviation. *p*-values < 0.05 were considered statistically significant.

## 3. Results

Demographics and basic clinical parameters of study subjects.

A total of 90 eyes of 90 PNV patients were included in this study. Of these, 50 showed a complete resolution of fluid after three aflibercept injections (dry group), and 40 had remaining fluid after treatment (non-dry group). The mean age was 61.5 ± 13.3 years, and 61 (67.8%) were male. Baseline and 3-month logMAR BCVAs were 0.337 ± 0.344 and 0.345 ± 0.367, respectively (*p* = 0.013).

The mean logMAR BCVA at 3 months was lower in the dry group compared to the non-dry group (0.175 ± 0.399 vs. 0.334 ± 0.306, *p* = 0.035). There was no difference in age, sex, laterality, or the presence of diabetes and hypertension between groups. The comparison of clinical characteristics between groups is summarized in Table 1.

### 3.1. Comparison of OCT Parameters between the Dry and Non-Dry Group

The mean CMT at 3 months was thicker (239.68 ± 65.81 μm vs. 297.83 ± 111.44 μm, *p* = 0.003), and the SFCT at baseline and 3 months was thinner (399.1 ± 60 μm vs. 342.13 ± 38.91 μm and 367.2 ± 67.35 μm vs. 321.53 ± 46.54 μm, respectively, both *p* < 0.001) in the non-dry group compared to the dry group. The mean CVD measured on OCT B-scan at baseline and 3 months was lower in the non-dry group compared to the dry group (0.768 ± 0.036 vs. 0.727 ± 0.039 μm and 0.76 ± 0.042 μm vs. 0.731 ± 0.038 μm, respectively, both *p* ≤ 0.001).

### 3.2. Comparison of UWICGA CVD between the Dry and Non-Dry Group

The CVD measured on UWICGA of the total visible area was 0.323 ± 0.034 in the dry group and 0.286 ± 0.038 in the non-dry group (*p* < 0.001). The intraclass correlation coefficient for CVD on UWICGA was 0.971 (95% confidence interval: 0.956–0.981) between two graders. This difference was also significant in each quadrant (*p* < 0.001, <0.001, =0.010, <0.001 for ST, SN, IN, and IT, respectively). The CVD of vortex ampullae was also higher in the dry group (0.416 ± 0.053 vs. 0.381 ± 0.063, *p* = 0.005) but did not remain significant when divided by the quadrant (all *p* ≥ 0.056), except for the IT quadrant (*p* = 0.034). The values are summarized in Table 2.

**Figure 2:**
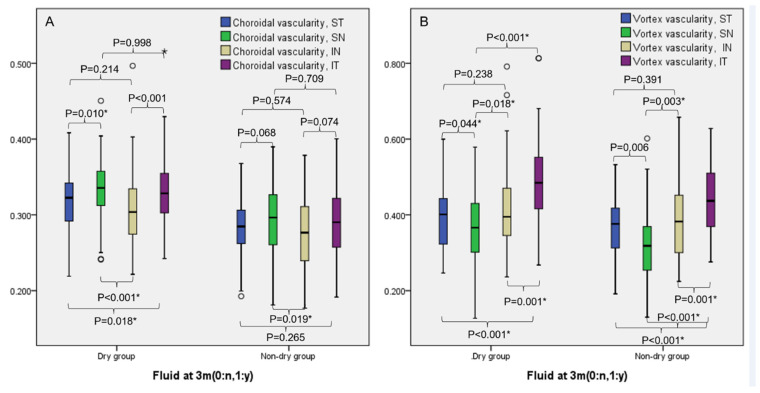
Comparison of choroidal vessel density (CVDs) of each quadrant measured on ultra-widefield indocyanine angiography between the dry and non-dry group. (**A**) CVDs of the superonasal (SN) and inferotemporal (IT) quadrants were higher than those of the superotemporal (ST) and inferonasal (IN) quadrants in both groups. (**B**) CVDs of the vortex ampullae were higher in the IT quadrant compared to other quadrants in both groups. * *p*-value < 0.05.

In both groups, the CVD of the SN and IT quadrants was higher than the ST and IN quadrants (all *p* ≤ 0.018, Figure 2A). The CVD of vortex ampullae was higher in the IT quadrant compared to other quadrants in both groups (all *p* ≤ 0.001, Figure 2B).

### 3.3. Analysis of Other Choroidal Vascular Features on UWICGA Images

**Figure 3:**
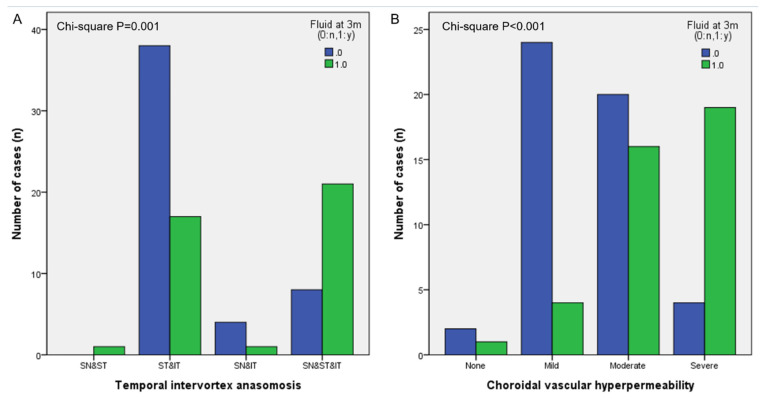
Analysis of temporal intervortex anastomosis and choroidal vascular hyperpermeability (CVH) grade on ultra-widefield indocyanine angiography images. (**A**) Superotemporal (ST) and inferotemporal (IT) vortex veins were the most common in intervortex anastomosis in the dry group, while anastomosis among three vortex veins, the superonasal (SN), ST and IT, was the most common in the non-dry group. (**B**) CVH grades differed between the dry and non-dry group. The mean CVH grade was 1.48 ± 0.735 in the dry group and 1.875 ± 0.822 in the non-dry group (*p* = 0.020).

Temporal and nasal intervortex anastomosis was observed in 96% and 56% of the dry group and 90% and 38% of the non-dry group, respectively (*p* = 0.257 and 0.081). The most common anastomosis at the temporal side was between ST and IT vortex veins in the dry group, while anastomosis among three vortex veins, the SN, ST and IT, was the most common in the non-dry group (*p* = 0.001, Figure 3A). The CVD of the total area was higher in eyes with two vortex anastomoses compared to three vortex anastomoses (0.315 ± 0.037 vs. 0.289 ± 0.041, *p* = 0.012). CVH was present in 96% of the dry group and 98% of the non-dry group (*p* = 0.694). The mean CVH grade was 1.48 ± 0.735 in the dry group and 1.875 ± 0.822 in the non-dry group (*p* = 0.020, Figure 3B). The CVH grade was lower in eyes with two vortex anastomoses compared to three vortex anastomoses (1.74 ± 0.87 vs. 2.17 ± 0.66, *p* = 0.020).

### 3.4. Correlation among Parameters

There were positive correlations among SFCT, CVD on B-scan, and CVD of the total area on UWICGA (all *p* ≤ 0.049, Table 3). When CVD on UWICGA was divided into quadrants, correlations were significant for the ST and SN quadrants. The IN quadrant correlated with CVD on B-scan (both *p* ≤ 0.009), and the IT quadrant correlated with baseline SFCT and baseline CVD on B-scan (both *p* ≤ 0.011). LogMAR BCVA showed a negative correlation with CVD on UWICGAs except for the IN quadrant (all *p* ≤ 0.020).

**Figure 4:**
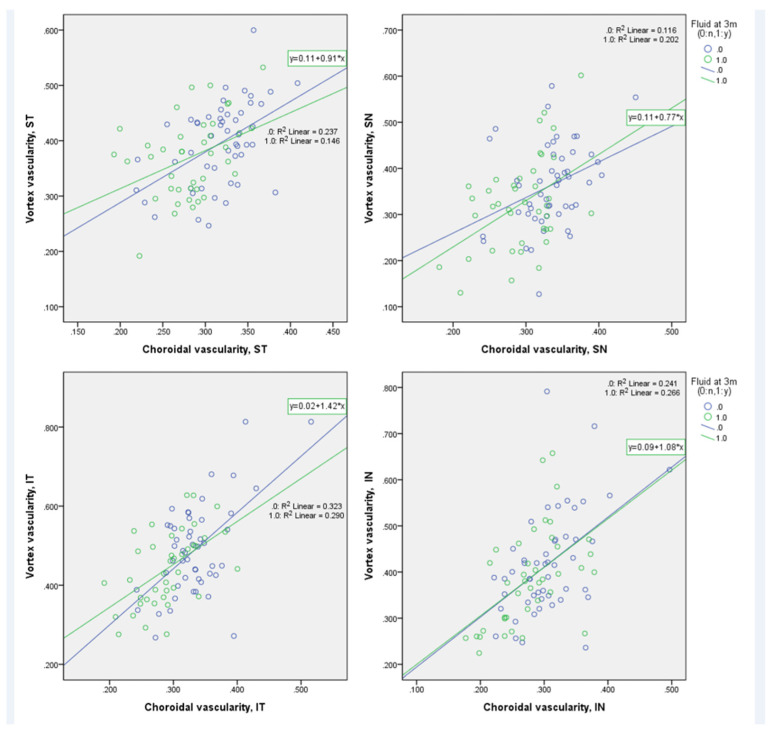
Correlation between choroidal vessel density (CVD) of each quadrant and vessel density around vortex ampullae of corresponding quadrants. In each quadrant, CVDs of vortex ampullae positively correlated with CVDs of the corresponding quadrants in both dry and non-dry groups.

In each quadrant, the CVD of vortex ampullae positively correlated with the CVD of the corresponding quadrants in both groups (in the dry group, r = 0.487, 0.341, 0.491, and 0.568, respectively, for ST, SN, IN, and IT, all *p* ≤ 0.015; in the non-dry group, r = 0.382, 0.449, 0.561, and 0.539, respectively, for ST, SN, IN, and IT, all *p* ≤ 0.015, Figure 4).

## 4. Discussion

In this study, we assessed the association between choroidal vascular morphology, especially the large vessels, in eyes with PNV and the short-term response to aflibercept treatment by categorizing PNV eyes into dry and non-dry groups after three injections. Quantitative differences in SFCT and CVD on OCT and UWICGA were found between the dry and non-dry groups. Other choroidal features, including the intervortex anastomosis constitution and CVH, also differed between groups.

Choroidal thickness has been reported as a prognostic factor after anti-VEGF treatment for nAMD and PCV [16]. In typical nAMD, SFCT was thinner among patients who achieved complete resolution of macular exudation [17]. However, in PCV, Kong et al. and Sakurada et al. demonstrated that eyes with a thin choroid manifested a worse visual function than eyes with a medium or thick choroid after three anti-VEGF treatments, and a higher SFCT was associated with a better visual outcome [18,19]. On the contrary, Chang et al. reported that PCV eyes with pachychoroid features showed less response to anti-VEGF treatment [20]. The controversy among studies might have been caused by a wide range of SFCT among PCV eyes [21]. In this study, PNV eyes with a thinner choroid were more likely to retain fluid after three aflibercept injections, which was similar to the findings by Kong et al. and Sakurada et al. These findings should be interpreted with caution, since treatment results can differ if the treatment duration is extended to 12 months [22]. Shin et al. reported that PCV eyes with thick choroids retained retinal fluid more often, while eyes with thin choroids experienced a more frequent resolution of retinal fluid from the baseline to 12 months after treatment.

The discordance between nAMD and PCV or PNV may be explained by differences in the underlying pathophysiology in these eyes. Drusen and choroidal thinning in nAMD represents a hypoxic condition, which is associated with the main pathophysiologic mechanism of the disease, while a thinner choroid in PCV and PNV eyes may result from choroidal atrophy in addition to what is presumably the main mechanism of choroidal vessel congestion and CVH in pachychoroid disease [23]. Kong et al. hypothesized that age-related choroidal atrophy accompanying PCV might decrease retinal function, which is associated with a low BCVA [18]. In accordance with the result showing that a thinner choroid was associated with a worse BCVA, lower CVD on UWICGA was associated with a worse baseline and 3-month BCVA in this study. Since age itself did not show a prominent correlation with CVD, there might be other good reasons for choroidal atrophy, such as chronicity of the disease.

The intervortex anastomosis status and CVH grade on UWICGA were significantly different between the dry and non-dry groups. Anastomosis between vortex veins of ST and IT quadrants was the most common form of temporal intervortex anastomosis in the dry group, whereas ST, ST, and IT vortex veins participated in temporal intervortex anastomosis in the non-dry group. A higher number of vortex veins in anastomosis may be indicative of a more chronic disease state. The lower CVD in eyes with a higher number of vortex veins participating in anastomosis can be explained by choroidal atrophy due to a chronic pathologic state. Not only was the number of vortex veins higher in the non-dry group, the CVH grade was higher compared to the dry group. CVH is a known prognostic factor for a poor response to anti-VEGF injections in PCV eyes [24]. Disease chronicity and a more defective choriocapillaris might have caused a poor response to anti-VEGF in the non-dry group.

There are several limitations to this study. First, it is retrospective in nature, had a relatively small number of patients from a single center, and included subjects from a single ethnicity. Second, this study did not include patients under the age of 50 due to limitations in national insurance reimbursement criteria for aflibercept use in the study country. In addition, this study was limited to eyes with an SFCT of 300 um or greater, but the cut-off value of 300 um is not an absolute criterion for defining pachychoroid, and there could be pachychoroid eyes with an SFCT under 300 um. Inclusion of these eyes may lead to different results, and further research regarding this is warranted. Finally, a longer follow-up period and other treatment-related parameters such as partial response or the required injection numbers should be considered in a different study. Nonetheless, this study has value as the first quantitative as well as qualitative analysis of choroidal vessel morphology on UWICGA and of the treatment response to aflibercept in eyes with PNV.

In summary, a poor short-term response to aflibercept treatment in PNV eyes was associated with a lower Haller vessel density, higher CVH grade, and intervortex anastomosis involving more quadrants on UWICGA. These findings provide insight into the pathophysiology and treatment response in pachychoroid eyes.

## Figures and Tables

**Table 1:** Comparison of clinical characteristics between dry and non-dry PNV groups.

Parameters	Dry Group (*n* = 50)	Non-Dry Group (*n* = 40)	*p*-Value
Age (mean ± SD, years)	59.74 ± 13.55	63.63 ± 12.91	0.169 ^a^
Sex (male, %)	62	75	0.190 ^b^
Laterality (left eye, %)	50	48	0.816 ^b^
Diabetes (n, (%))	18	15	0.706 ^b^
Hypertension (n, (%))	44	48	0.744 ^b^
BCVA, baseline (mean ± SD, logMAR)	0.175 ± 0.399	0.334 ± 0.306	0.035 ^a,^*

PNV: pachychoroid neovasculopathy; SD: standard deviation; BCVA: best-corrected visual acuity; logMAR: logarithm of minimal angle of resolution. ^a^: Chi-square test. ^b^: Mann–Whitney U-test. * Statistically significant *p*-value.

**Table 2:** Comparison of quantitative choroidal features between dry and non-dry pachychoroid neovasculopathy groups.

Parameters	Dry Group (*n* = 50)	Non-Dry Group (*n* = 40)	*p*-Value
CMT, baseline (μm)	316.84 ± 118.22	367.03 ± 185.21	0.142
CMT, 3 m (μm)	239.68 ± 65.81	297.83 ± 111.44	0.005 *
SFCT, baseline (μm)	399.1 ± 60	342.13 ± 38.91	<0.001 *
SFCT, 3 m (μm)	367.2 ± 67.35	321.53 ± 46.54	<0.001 *
CVD, Bscan, baseline	0.768 ± 0.036	0.727 ± 0.039	<0.001 *
CVD, Bscan, 3-months	0.760 ± 0.042	0.731 ± 0.038	0.001 *
CVD, UWICGA, total	0.323 ± 0.034	0.286 ± 0.038	<0.001 *
CVD, UWICGA, ST	0.317 ± 0.040	0.283 ± 0.04	<0.001 *
CVD, UWICGA, SN	0.334 ± 0.040	0.293 ± 0.045	<0.001 *
CVD, UWICGA, IN	0.306 ± 0.051	0.278 ± 0.052	0.010 *
CVD, UWICGA, IT	0.334 ± 0.047	0.290 ± 0.046	<0.001 *
CVD, vortex ampullae, total	0.416 ± 0.053	0.381 ± 0.063	0.005 *
CVD, vortex ampullae, ST	0.395 ± 0.075	0.370 ± 0.073	0.120
CVD, vortex ampullae, SN	0.363 ± 0.091	0.323 ± 0.102	0.056
CVD, vortex ampullae, IN	0.418 ± 0.113	0.387 ± 0.106	0.187
CVD, vortex ampullae, IT	0.490 ± 0.117	0.442 ± 0.094	0.034 *

CMT: central macular thickness; SFCT: subfoveal choroidal thickness; CVD: choroidal vessel density; UWICGA: ultra-widefield indocyanine green angiogaphy; ST: superotemporal; SN: superonasal; IN: inferonasal; IT: inferotemporal. * Statistically significant *p*-value.

**Table 3:** Correlation between clinical parameters in pachychoroid neovasculopathy.

Clinical Parameters		Choroidal Vessel Densities on UWICGA				
		Total	ST	SN	IN	IT
Age	Coefficient	−0.174	−0.184	−0.226 *	−0.100	−0.074
	*p*-value	0.100	0.082	0.032	0.347	0.490
LogMAR BCVA, baseline	Coefficient	−0.291 **	−0.385 **	−0.245 *	−0.090	−0.261 *
	*p*-value	0.005	0.000	0.020	0.400	0.013
LogMAR BCVA, 3 months	Coefficient	−0.351 **	−0.468 **	−0.265 *	−0.188	−0.256 *
	*p*-value	0.001	0.000	0.012	0.076	0.015
CMT, baseline	Coefficient	−0.015	−0.081	−0.033	0.044	0.006
	*p*-value	0.887	0.449	0.760	0.684	0.954
CMT, 3 months	Coefficient	−0.080	−0.043	−0.092	−0.048	−0.078
	*p*-value	0.455	0.689	0.390	0.657	0.466
SFCT, baseline	Coefficient	0.353 **	0.374 **	0.400 **	0.140	0.268 *
	*p*-value	0.001	0.000	0.000	0.187	0.011
SFCT, 3 months	Coefficient	0.247 *	0.350 **	0.291**	0.038	0.164
	*p*-value	0.019	0.001	0.005	0.721	0.123
CVD, Bscan, baseline	Coefficient	0.480 **	0.425 **	0.423 **	0.362 **	0.365 **
	*p*-value	0.000	0.000	0.000	0.000	0.000
CVD, Bscan, 3 months	Coefficient	0.315 **	0.356 **	0.273 **	0.216 *	0.199
	*p*-value	0.003	0.001	0.009	0.040	0.060

UWICGA: ultra-widefield indocyanine green angiogaphy; ST: superotemporal; SN: superonasal; IN: inferonasal; IT: inferotemporal; LogMAR: logarithm of minimal angle of resolution; BCVA: best-corrected visual acuity; CMT: central macular thickness; SFCT: subfoveal choroidal thickness; CVD: choroidal vessel density. * Significant correlation at the 0.05 level by Pearson’s correlation. ** Significant correlation at the 0.01 level by Pearson’s correlation.

## Data Availability

The datasets generated and/or analyzed during the current study are available from the corresponding author upon reasonable request.

## References

[1] Pang C.E., Freund K.B. (2015). Pachychoroid Neovasculopathy. Retina.

[2] Cheung C.M.G., Lai T.Y.Y., Ruamviboonsuk P., Chen S.J., Chen Y., Freund K.B., Gomi F., Koh A.H., Lee W.K., Wong T.Y. (2018). Polypoidal Choroidal Vasculopathy: Definition, Pathogenesis, Diagnosis, and Management. Ophthalmology.

[3] Warrow D.J., Hoang Q.V., Freund K.B. (2013). Pachychoroid pigment epitheliopathy. Retina.

[4] Elfandi S., Ooto S., Miyata M., Ueda-Arakawa N., Subhi Y., Yamashiro K., Tamura H., Oishi A., Hata M., Yoshimura N. (2021). Effects of Intravitreous Aflibercept Injection in Pachychoroid Neovasculopathy: Comparison with Typical Neovascular Age-Related Macular Degeneration. Clin. Ophthalmol..

[5] Jung B.J., Kim J.Y., Lee J.H., Baek J., Lee K., Lee W.K. (2019). Intravitreal aflibercept and ranibizumab for pachychoroid neovasculopathy. Sci. Rep..

[6] Matsumoto H., Hiroe T., Morimoto M., Mimura K., Ito A., Akiyama H. (2018). Efficacy of treat-and-extend regimen with aflibercept for pachychoroid neovasculopathy and Type 1 neovascular age-related macular degeneration. Jpn. J. Ophthalmol..

[7] Baek J., Lee J.H., Jeon S., Lee W.K. (2019). Choroidal morphology and short-term outcomes of combination photodynamic therapy in polypoidal choroidal vasculopathy. Eye.

[8] Azuma K., Okubo A., Nomura Y., Zhou H., Terao R., Hashimoto Y., Asano K.S., Azuma K., Inoue T., Obata R. (2020). Association between pachychoroid and long-term treatment outcomes of photodynamic therapy with intravitreal ranibizumab for polypoidal choroidal vasculopathy. Sci. Rep..

[9] Karasu B., Akbas Y.B., Kaskal M., Aykut A., Celebi A.R.C. (2022). Long term results of three anti-vascular endothelial growth factor agents in pachychoroid neovasculopathy. Cutan. Ocul. Toxicol..

[10] Azuma K., Tan X., Asano S., Shimizu K., Ogawa A., Inoue T., Murata H., Asaoka R., Obata R. (2019). The association of choroidal structure and its response to anti-VEGF treatment with the short-time outcome in pachychoroid neovasculopathy. PLoS ONE.

[11] Hara C., Wakabayashi T., Toyama H., Fukushima Y., Sayanagi K., Sato S., Sakaguchi H., Nishida K. (2018). Characteristics of patients with neovascular age-related macular degeneration who are non-responders to intravitreal aflibercept. Br. J. Ophthalmol..

[12] Spaide R.F. (2018). Disease expression in nonexudative age-related macular degeneration varies with choroidal thickness. Retina.

[13] Spaide R.F., Koizumi H., Pozonni M.C. (2008). Enhanced Depth Imaging Spectral-Domain Optical Coherence Tomography. Am. J. Ophthalmol..

[14] Lee A., Ra H., Baek J. (2020). Choroidal vascular densities of macular disease on ultra-widefield indocyanine green angiography. Graefes Arch. Clin. Exp. Ophthalmol..

[15] Spaide R.F., Ledesma-Gil G., Gemmy Cheung C.M. (2021). Intervortex venous anastomosis in pachychoroid-related disorders. Retina.

[16] Jirarattanasopa P., Ooto S., Nakata I., Tsujikawa A., Yamashiro K., Oishi A., Yoshimura N. (2012). Choroidal thickness, vascular hyperpermeability, and complement factor H in age-related macular degeneration and polypoidal choroidal vasculopathy. Investig. Ophthalmol. Vis. Sci..

[17] Kim H., Lee S.C., Kwon K.Y., Lee J.H., Koh H.J., Byeon S.H., Kim S.S., Kim M., Lee C.S. (2016). Subfoveal choroidal thickness as a predictor of treatment response to anti-vascular endothelial growth factor therapy for polypoidal choroidal vasculopathy. Graefes Arch. Clin. Exp. Ophthalmol..

[18] Kong M., Kim S.M., Ham D.I. (2017). Comparison of clinical features and 3-month treatment response among three different choroidal thickness groups in polypoidal choroidal vasculopathy. PLoS ONE.

[19] Sakurada Y., Sugiyama A., Tanabe N., Kikushima W., Kume A., Iijima H. (2017). Choroidal thickness as a prognostic factor of photodynamic therapy with aflibercept or ranibizumab for polypoidal choroidal vasculopathy. Retina.

[20] Chang Y.C., Cheng C.K. (2019). Difference between Pachychoroid and Nonpachychoroid Polypoidal Choroidal Vasculopathy and Their Response to Anti-Vascular Endothelial Growth Factor Therapy. Retina.

[21] Lee W.K., Baek J., Dansingani K.K., Lee J.H., Freund K.B. (2016). Choroidal morphology in eyes with polypoidal choroidal vasculopathy and normal or subnormal subfoveal choroidal thickness. Retina.

[22] Shin J.Y., Kwon K.Y., Byeon S.H. (2015). Association between choroidal thickness and the response to intravitreal ranibizumab injection in age-related macular degeneration. Acta Ophthalmol..

[23] Spaide R.F. (2009). Age-related choroidal atrophy. Am. J. Ophthalmol..

[24] Miyake M., Tsujikawa A., Yamashiro K., Ooto S., Oishi A., Tamura H., Nakata I., Matsuda F., Yoshimura N. (2014). Choroidal neovascularization in eyes with choroidal vascular hyperpermeability. Investig. Ophthalmol. Vis. Sci..

